# Chemotherapy and palliative care near end-of life: examining the appropriateness at a cancer institute for colorectal cancer patients

**DOI:** 10.1186/s12904-018-0339-8

**Published:** 2018-06-19

**Authors:** Ilaria Massa, Oriana Nanni, Flavia Foca, Marco Maltoni, Stefania Derni, Nicola Gentili, Giovanni Luca Frassineti, Andrea Casadei Gardini, Martina Valgiusti, Dino Amadori, Elena Prati, Mattia Altini, Davide Gallegati, Elisabetta Sansoni

**Affiliations:** 1 Unit of Biostatistics and Clinical Trials, Istituto Scientifico Romagnolo per lo Studio e la Cura dei Tumori (IRST) IRCCS, Meldola, Italy; 2 Palliative Care Unit, Istituto Scientifico Romagnolo per lo Studio e la Cura dei Tumori (IRST) IRCCS, Meldola, Italy; 3 Palliative Care Unit, AUSL Romagna, Forlì, Italy; 4 IT Unit, Istituto Scientifico Romagnolo per lo Studio e la Cura dei Tumori (IRST) IRCCS, Meldola, Italy; 5 Department of Medical Oncology, Istituto Scientifico Romagnolo per lo Studio e la Cura dei Tumori (IRST) IRCCS, Meldola, Italy; 6 Healthcare Administration, Istituto Scientifico Romagnolo per lo Studio e la Cura dei Tumori (IRST) IRCCS, Meldola, Italy; 7 Planning and Control, Istituto Scientifico Romagnolo per lo Studio e la Cura dei Tumori (IRST) IRCCS, Meldola, Italy

**Keywords:** Appropriateness, Chemotherapy, Palliative care, End of life, Indicators

## Abstract

**Background:**

Appropriate cessation of chemotherapy and timely referral of patients to hospice services are crucial for the quality of care near death. We investigated the quality of care in our Cancer Institute in very advanced metastatic colorectal cancer patients treated in real life.

**Patients and Methods:**

We performed a retrospective analysis of electronic medical data of patients with metastatic colorectal cancer who were candidates for chemotherapy during the study period (1 January 2007–30 June 2014) and died before 31 December 2014. Quality-of-cancer-care indicators were calculated for the overuse of chemotherapy and referral to hospice. Predictive factors of chemotherapy discontinuation and hospice referral in end-of life care were investigated using parametric and nonparametric methods.

**Results:**

Of the 365 patients who died before 31 December 2014, 26 (7.1%) received chemotherapy in the last 14 days of life and 36 (9.8%) started a new chemotherapy regimen in the last 30 days of life. Factors associated with the overuse of chemotherapy were being < 70 years of age for both indicators and not having received advanced chemotherapy treatments for the former indicator. The majority of patients (74.7%) had access to hospice services, of whom only a small percentage (7.2%) accessed them very near to death.

**Conclusions:**

According to the criteria used, our Institute provides a good quality of cancer care for dying colorectal cancer patients, measured by the use of chemotherapy and referral to hospice in their last days of life.

## Background

Despite the progress made in cancer care, cancer is still a lethal disease and the use of chemotherapy remains crucial for both curative and palliative purposes. However, the appropriate use of chemotherapy is still unclear near death. The subtle difference between curative and palliative intent when palliative chemotherapy can extend life is under debate [1]. In addition, patient prognosis is complex to predict and the few survival prediction tools available are not easy to use. To date, there has been a lack of international guidelines on the management of cancer patients near death, except for the American Society of Clinical Oncology (ASCO) and the European Society of Medical Oncology [2, 3]. Therefore, the decision to stop a specific treatment in favor of the best palliative care is still incredibly hard and the so-called “therapeutic inertia”, i.e. the persistence of inappropriate antitumor treatments in non-responding terminal patients, is frequently used. Such overly aggressive care not only affects a patient’s quality of life (QoL) [4] but, from a healthcare system point of view, it raises questions about system sustainability given the increasing costs of cancer care (about 20% per year in the U.S. in the period 1990–2020) [5].

Earle et al. identified aggressive care near the end of life (EoL) as a sign of poor quality cancer services [6] and proposed quality indicators of cancer care for external benchmarking. Three major areas were evaluated: a) possible misuse of treatment resulting in high rates of emergency room visits, hospitalization, or intensive care unit admission; b) lack of referral or very late referral to hospice; c) overuse of chemotherapy very near to death [7]. Although numerous studies have measured some of these indicators, different criteria were used to define populations of interest, making a comparison of results more difficult.

In the present paper we report our data from a retrospective cohort study conducted at our institute (IRST IRCCS) in which we assessed the behavior of physicians towards chemotherapy prescription and the access of terminal colorectal cancer (CRC) patients to hospice care. Our objectives were to gain better knowledge have a better knowledge of the quality of cancer care services in our institute, and a deeper insight into how we manage end-of-life EoL care in a subset of patients. In particular, we aimed to report the frequency of some internationally acknowledged indicators of quality cancer care and to identify whether some factors related to patient characteristics or the organization of services provided can affect the use of chemotherapy and the referral to hospice care at EoL.

## Methods

### Study design and participants

We conducted a retrospective analysis of the electronic medical data of metastatic CRC patients with candidate to receive chemotherapy. We included all the consecutive patients resident in the catchment area of Forlì (188,357 inhabitants ref. year 2013) and in the adjacent area of Cesena Local Health Authority (AUSL) (209,622 inhabitants ref. year 2013) who were candidates to receive chemotherapy in a metastatic setting from 1 January 2007 to 30 June 2014 and had died before 31 December 2014. The following information was collected for each patient: age of death, gender, number of lines received for advanced disease, date of last cycle of chemotherapy and date of start of last new line of therapy. ECOG Performance Status information was collected on the day of the last chemotherapy line prescription and on the day of the last administration, or in a time range varying from the day of the last administration and 7 days before administration.

With regard to access to palliative care, analysis was possible only for a subgroup of patients who were resident in the Forlì catchment area and who died between February 2009 and December 2014, since registration of access to the palliative care network was recorded from February 2009 onwards. As cancer patients living in the two neighboring areas of Forlì and Cesena are followed by our cancer institute, we were able to verify the homogeneity of the two populations by gender, age, previous treatment for advanced disease, PS ECOG (0 vs. > 1) (data not shown).

The date of the first access to palliative care was retrieved for patients admitted to hospice or home-based hospice care for at least 1 day. Results obtained for this subpopulation can be generalized are representative of the entire population because patient characteristics were the same and all patients were followed at our institute. Patients who had only received best supportive care and patients enrolled in clinical trials were excluded.

### Description of indicators

To evaluate appropriate prescription of chemotherapy, we considered 2 indicators proposed by Earle et al. [7] and measured them on the population of patients receiving chemotherapy, as follows:

- proportion of patients who started a new chemotherapy regimen in the last 30 days of life. The proposed appropriate threshold of < 2% was set as standard.
- proportion of patients who received chemotherapy administration in the last 14 days of life.

The proposed appropriate threshold of < 10% was set as standard by Earle et al. [7].

We considered all types of palliative care interventions which represent constitute the “Local Palliative Care Network (LPCN)”. The network is divided into two types of interventions: early palliative care, which is further divided into outpatient and inpatient care for patients undergoing chemotherapy, and EoL palliative care, which is further divided into inpatient hospice care and home-based hospice care mainly for those who are no longer amenable to chemotherapy. Although some authors [8] consider home-based hospice care as a form of early palliative care, we, together with others [9–11], regard it as a form of EoL palliative care, as the average length of stay for patients in this setting is about 60–90 days.

With regard to early palliative care interventions during the study period, oncologists did not systematically refer patients to a palliative care specialist but only requested their intervention for pain and symptom management. We did not thus were unable to analyze the impact of early palliative care on the decision of the oncologist as to whether or not prescribe administer chemotherapy.

We used the following 2 indicators proposed by Earle et al. [7], measured on the population of patients for whom the date of access to palliative care was available, to evaluate appropriate access to hospice services:

- proportion of patients receiving hospice services before death to compare with the proposed appropriate threshold of > 55%;
- proportion of the above-mentioned patients admitted to hospice 3 days before death to compare with the proposed appropriate threshold of < 8%.

The proposed appropriate thresholds were set as standard by Earle et al. [7].

We then compared our data considering both forms of EoL palliative care.

### Statistical methods

Categorical variables were expressed as frequencies and percentages, while continuous variables were presented using median and range.

The relationship between demographic and clinical characteristics and patient status based on the considered indicators were analyzed using chi-square, Fisher exact test or Cochran-Armitage test for trend as appropriate. In case of continuous variables, Wilcoxon Mann-Whitney test was used.

Logistic regression was used to investigate the effect explanatory variables (age, gender and previous line of advanced therapy) to evaluate the physician’s behavior in the management of cancer patients in the last days of life (last chemotherapy scheme prescription ≤30 days vs. > 30 days to death; last chemotherapy cycle administration ≤14 days vs. > 14 days to death). To describe the association, adjusted odds ratios (OR) with 95% profile likelihood confidence intervals (95%CI) were evaluated. A *p*-value of < 0.05 was considered statistically significant. Statistical analyses were carried out with SAS Statistical software (version 9.3, SAS Institute, Cary, NC, USA).

## Results

### Population characteristics

We observed 365 patients with metastatic CRC deceased before 31 December 2014 who had started a new chemotherapy regimen with palliative intent in at our Institute between 1 January 2007 and 30 June 2014. Males were 58.9% and median age was 70 years (33–87) (Table 1). Considering previous treatment features, 245 patients out of 365 (67.1%) had already received at least 1 chemotherapy treatment in metastatic setting.

**Table 1:** Patient characteristics and factors associated to the use of CT in the EoL

	Total No. (%)	New CT scheme prescription > 30 days to death No. (%)	New CT scheme prescription ≤30 days to death No. (%)	*p*-value	Last CT cycle administration > 14 days to death No. (%)	Last CT cycle administration ≤14 days to death No. (%)	*p*-value
Overall	365 (100.0)	329 (90.1)	36 (9.8)	–	339 (92.9)	26 (7.1)	–
Gender							
Male	215 (58.9)	190 (88.4)	25 (11.6)	0.18	200 (93.0)	15 (7.0)	0.90
Female	150 (41.1)	139 (92.7)	11 (7.3)		139 (92.7)	11 (7.3)	
Age							
Median (range)	70 (33–87)	71 (33–87)	63.5 (37–83)	0.13	70 (33–87)	62.5 (45–80)	0.09
≤ 70 years	188 (51.5)	164 (87.2)	24 (12.8)	0.05	170 (90.4)	18 (9.6)	0.06
> 70 years	177 (48.5)	165 (93.2)	12 (6.8)		169 (95.5)	8 (4.5)	
Period of study inclusion							
2007–2010	142 (38.9)	129 (90.9)	13 (9.1)	0.72	136 (95.8)	6 (4.2)	0.09
2011–2014	223 (61.1)	200 (89.7)	23 (10.3)		203 (91.0)	20 (8.9)	
Previous treatment for advanced disease							
None	120 (32.9)	108 (90.0)	12 (10.0)	0.95	105 (87.5)	15 (12.5)	< 0.01
At least one	245 (67.1)	221 (90.2)	24 (9.8)		234 (95.5)	11 (4.5)	

### Use of chemotherapy

Thirty-six (9.8%) patients out of 365 started a new chemotherapy regimen in the last month of life, while 26 (7.1%) patients received last chemotherapy administration within 14 days before death. Out of the former 36 patients receiving a new chemotherapy regimen, 12 (33.3%) received a first-line treatment, 12 (33.3%) a second-line, 8 (22.2%) a third-line, and 4 (11.2%) > third-line.

**Fig. 1:**
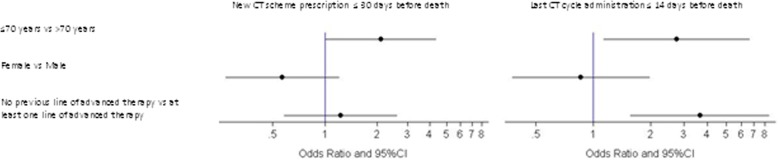
Clinical factors associated to therapeutic inertia

Results showed that, among the patients receiving aggressive chemotherapy approach in the last month of life, patients aged ≤70 years of age were likely to receive a more aggressive approach (12.8% vs. 6.8%, *p* = 0.05) than older patients. Considering the last chemotherapy administration in the last 14 days of life, age ≤ 70 years (9.6% vs. 4.5%, *p* = 0.06) and not having received previous advanced treatments (*p* < 0.01) were predictive factors for aggressive behavior. Neither gender nor years of treatment (2007–2010 vs. 2011–2014) appeared to influence the chemotherapy approach (Table 1). These results were maintained in the multivariate analysis (Fig. 1).

With regard to ECOG Performance Status (PS) at the start of the last line of chemotherapy (or within the previous 7 days) (data were available for 192 patients out of 365, 52.6%), we observed that 158 (82.3%) patients had either ECOG PS 0 or 1, while 34 patients (17.7%) had either ECOG PS 2 or 3 (Table 2). At the time of the last chemotherapy administration, ECOG PS was available for 129 patients out of 365 (35.3%) and was either 0 or 1 for 91 patients (70.6%), and either 2 or 3 for 38 patients (29.4%). As expected, we found that the distribution of PS of patients who received both a new chemotherapy line in the last month of life and the last chemotherapy administration in the last 14 days of life and those who did not, was different, worsening for the group of patients who received chemo in the last period of life. This factor was not included in the multivariate analysis due to the high amount of missing data. These results are maintained in a multivariate analysis (Fig. 1).

**Table 2:** EoL chemotherapy and PS ECOG distribution

	Total No. (%)	New CT scheme prescription > 30 days to death No. (%)	New CT scheme prescription ≤30 days to death No. (%)	*p*-value	Total No. (%)	Last CT cycle administration > 14 days to death No. (%)	Last CT cycle administration ≤14 days to death No. (%)	*p*-value
Overall	192 (100.0)	170 (88.5)	22 (11.5)		129 (100.0)	115 (89.1)	14 (10.9)	
PS (ECOG)								
0	78 (40.6)	75 (96.2)	3 (3.8)	< 0.01	34 (26.4)	32 (94.1)	2 (5.9)	0.01
1	80 (41.7)	74 (92.5)	6 (7.5)		57 (44.2)	51 (89.5)	6 (10.5)	
2	28 (14.6)	18 (64.3)	10 (35.7)		35 (27.1)	30 (85.7)	5 (14.3)	
3	6 (3.1)	3 (50.0)	3 (50.0)		3 (2.3)	2 (66.7)	1 (33.3)	

### Access to hospice services

Regarding access to LPCN, we found that out of 166 patients resident in the Forlì catchment Local health Authority area, 28 (16.9%) patients had never had a palliative care contact with the LPCN in the advanced setting, while the remaining 138 (83.1%) had had at least one contact. In particular, 73 (44%) patients had at least one early palliative care visit, 124 (74.7%) patients accessed at least one EoL palliative care service (Hospice inpatient admission and Home-Care Hospice Program), and 59 (35.5%) patients accessed both EoL palliative care services. Out of the 124 patients who had access to EoL palliative care, 9 (7.2%) referred to the hospice in the last 3 days of life (Table 3). A higher, albeit not significant, percentage of patients who had had an early palliative consultation (80.8% vs. 69.9%, *p* = 0.11) accessed the hospice. However, no difference in terms of appropriate access (> 3 days from death) to Hospice itself was found among patients.

**Table 3:** Relationship between early PC consultation and access to EoL-PC (*n* = 166)

	Total	Access to EoL Palliative Care			
		None	At least one	Before ≤3 days to death	Within ≤3 days to death
	No. (%)	No. (%)	No. (%)	No. (%)	No. (%)
Overall	166 (100.0)	42 (25.3)	124 (74.7)	115 (92.8)	9 (7.2)
Access to early PC consultation					
None	93 (56.0)	28 (30.1)	65 (69.9)		
At least one	73 (44.0)	14 (19.2)	59 (80.8)		

## Discussion

According to Earle’s criteria, our Institution carries out an acceptable “therapeutic inertia” in terms of chemotherapy administration in the last 14 days of life (7.1% compared to < 10% proposed by Earle). Nevertheless, improvements should be made concerning the start of a new chemotherapy regimen in the last month of life (9.8% compared to < 2% proposed by Earle). When considering factors predictive of therapeutic inertia, we found that age ≤ 70 years was associated with a more aggressive treatment in both starting a new chemotherapy line in the last 30 days of life and receiving chemotherapy in the last 14 days of life, as confirmed in literature [12–14]. Although this behavior might be acceptable from a psychological point of view, it is not indeed supported by any scientific basis, as highlighted by earlier studies [13]: a more balanced approach is currently being studied.

In this study, female gender and treating physician were no predictor factors, unlike in other studies [15]. Since our Institution is organized into pathology-specific medical teams, an appointed group of physicians regularly meeting to discuss clinical cases is in charge of the management of CRC patients, thus reducing bias of treating physician.

As for the role of PS in the decision making process, we observed that it is likely to be poorer for patients very near death. In fact, as expected, PS is poorer in patients receiving chemotherapy in the last period of life than in patients not receiving any, due to the different timing respect to death in which it was measured.

In clinical studies with chemotherapy, eligibility criteria usually couple a good PS (ECOG 0–1, less frequently 0–2) with a survival prognosis of at least 3 months. Functional activity indexes can correctly evaluate disability, but need to be combined and integrated with other parameters in order to assess prognosis. Recent evidence from Prigerson [4], that combined PS values, chemotherapy administration and QoL near death, showed that chemotherapy administration did not improve QoL for patients with poor PS. On the contrary, it can be detrimental on QoL for patients with good PS. Prognosis in advanced cancer patients is a highly debated topic. For this reason, some tools have been studied in palliative care. Among them, the Palliative Prognostic Score (PaP Score) [16–18] has been an accurate tool in several independent terminally ill cancer case series, classifying the studied population into 3 subgroups according to life expectancy and demonstrating that it could help clinicians to better select those patients who could actually benefit from a palliative chemotherapy. Pap Score could be assessed at every cycle of chemotherapy together with ECOG PS.

Some methodological issues on therapeutic inertia near death are noteworthy. Many publications [12, 19, 20] have measured these indicators in different types of cancer and settings in a non-homogeneous way, changing criteria to define the population of interest, so that it is almost impossible to compare data from different institutions.

We could compare our population, representing the entire population in the catchment area of our Cancer Institute, with a Canadian study [21] and an Australian study [15]: our percentage for treatment in the last 14 days is lower than in Canada, while it is similar to Australia. It is worth mentioning that, like in Australia but unlike in Canada, we restricted our analysis to CRC patients, excluding hematological malignancies which are usually more aggressively treated. In Earle’s publications, indicators were evaluated on a cohort monitored by one of the SEER (Surveillance, Epidemiology and End Results) registry whose Medicare population was > 65 years old, thus not allowing for wide comparability of percentages.

Focusing on “EoL palliative care interventions” within LPCN, we found that about 74.7% of our patients that died of cancer had received EoL palliative care. Overall, as only 16.9% of colorectal cancer patients had no access to LPCN during their cancer history, palliative care was guaranteed to the vast majority of the cancer population in this setting, and referring to EoL palliative care services, they were accessed in a timely and appropriate manner. We found also that referral to early palliative care consultation can aid subsequent referral to EoL hospice care.

In order to better understand the role of early palliative care (EPC) on patient outcome and QoL, our Institute carried out a randomized clinical trial on patients with advanced pancreas cancer, confirming that systematic EPC significantly improved QoL with respect to on-demand EPC. A positive impact was also observed for some indicators of EoL treatment aggressiveness, including the administration of chemotherapy during the last 30 days of life [22, 23]. These data confirmed the findings of Temel, Bakitas and ASCO Provisional Clinical Opinion, all of which highlighted the benefits of palliative care combined with standard anticancer therapies [24–26].

The main limitations of the present study include its retrospective design and the collection of data performed in a wide time range in a non-homogeneous way leading to a suboptimal completeness of retrieved data (i.e. ECOG PS). Moreover, as it was impossible to establish the reasons for chemotherapy discontinuation, we assumed it was due to progression of disease. Another limitation is that we hypothesized that death had always been cancer-related without, however, linking data to a death registry, which may have resulted in an underestimation of deaths. Despite these limitations, we used real-world clinical data, as the best informative way that can change clinical practice. An essential tool for this research was the Electronic Health Records (EHR), which allow to conduct a variety of research projects, from retrospective studies to monitoring programs like this one, that involve all treated patients for estimating the value of healthcare. Despite the lengthy adoption time of EHRs, we believe that data collection will improve over time both quantitatively and qualitatively, overcoming the limitations of using administrative data only. Our Institute has recently set up an operative taskforce, involving clinicians, methodologists, cost management specialists, and the healthcare direction to regularly verify clinical outcomes, appropriateness and costs, so that health resources can be employed to the best.

## Conclusions

In conclusion, our assessment of the frequency of some indicators of cancer care quality shows that, according to the criteria used, our institute provides a good quality of care to the CRC patients included in this study. For the Palliative Care Community, our experience can contribute to the “culture of measuring” that needs to be enhanced in EoL care, in order to improve the quality of care. In this vision, multicenter experiences must be promoted, including also cost evaluations for a more complete picture of EoL cancer care.

## Abbreviations

ASCO: American Society of Clinical Oncology
AUSL: Local Health Authority
CI: Confidence interval
ECOG PS: Eastern cooperative oncology group performance status
EHR: Electronic Health Record
EoL: End of life
EPC: Early palliative care
IRST IRCCS: Istituto Scientifico Romagnolo per lo Studio e la Cura dei Tumori (IRST) IRCCS
LPCN: Local Palliative Care Network
OR: Odds ratio
PaP Score: Palliative prognostic score
QoL: Quality of life
SEER: Surveillance Epidemiology and End Results
